# Acute Poisoning among Patients Presenting to the Emergency Department of a Tertiary Care Center: A Descriptive Cross-sectional Study

**DOI:** 10.31729/jnma.4997

**Published:** 2020-07-31

**Authors:** Sameer Thapa, Bishwa Raj Dawadi, Anup Raj Upreti

**Affiliations:** 1 Department of Emergency and General Practice, Nepal Medical College and Teaching Hospital, Attarkhel, Kathmandu, Nepal; 2 Department of Emergency and General Practice, Grande International Hospital, Dhapasi, Kathmandu, Nepal; 3 Clinical Pharmacist, Nepal Medical College and Teaching Hospital, Attarkhel, Kathmandu, Nepal

**Keywords:** *acute*, *emergency*, *organophosphates*, *poisoning*, *suicide*

## Abstract

**Introduction:**

Acute poisoning is a major global public health problem contributing to one of the leading causes for a visit to an emergency department. This study aims to analyse the demographic and psychosocial characteristics of patients with acute poisoning presented to the emergency department.

**Methods:**

This was a descriptive cross-sectional study conducted in a tertiary care hospital from June to December 2019 after obtaining ethical approval from Institutional review board (reference number. 041-075/0760). A convenient sampling method was applied. Epidemiological factors, types of poison consumed, reason, motive, and place to take poison, time elapse in the presentation to the hospital were studied. Statistical analysis was done using statistical package for the social sciences version 20. Point estimate at 95% Confidence Interval was calculated along with frequency and proportion for binary data.

**Results:**

Out of 76 cases of acute poisoning, the organophosphorus poisoning was 18 (23.7%) followed by unknown 12 (15.8). Of total, 28 (36.8%) had quarrel before taking poison and 41 (53.9%) had intention to commit suicide. Sixty-seven (88.2%) took a poison at home. The average elapsed time to the visit of the emergency department was 110±80 minutes

**Conclusions:**

The most common poisoning was organophosphorus with a suicide being the most common intention. Quarrel was the most frequent reason to take poison and the home was the most common place to take poison.

## INTRODUCTION

Acute poisoning is a significant global public health problem^1,2^ causing significant mortality and morbidity throughout the world.^3^ It also contributes to one of the leading causes for a visit to an emergency department.^4,5^

Evaluation of the socio-epidemiological factors of patients with acute poisoning presented to emergency departments such as geography, cultural and religious practices, profession, socioeconomic status, literacy rate support to develop tools to initiate preventive measures, and formulate practical guidelines in the management of acute poisoning in emergency departments.

In this study, we have tried to study about the demographic and psychosocial characteristics of patients with acute poisoning presented to emergency department.

## METHODS

This descriptive cross-sectional study was conducted in Nepal Medical College Private Limited and Teaching Hospital (NMCTH) for a period of six months (June to December, 2019). Ethical clearance was obtained from the Nepal Medical College Institutional Review Committee (reference number. 041-075/0760). Acute poisoning patients presenting to the Emergency Department were enrolled in the study (n = 76). All cases of acute poisoning, irrespective of age, sex, type and mode of poisoning, ingredients of poisons, and the status of patients after poisoning were included in the study. The patients included in the study were those who had undergone exposure to poison either by household or agricultural pesticides, stings bite, snake bite, industrial toxins, toxic plants, drug, or miscellaneous products. People from whom history could not be obtained and those not willing to participate were exclusion criteria in this study.

The sample size was calculated as,

$$\begin{array}{ll} \text{n} & \text{= }{\text{Z}}^{\text{2}}\times \text{p}\times \text{q}/e^{\text{2}}\\ & \text{= }{(1.96\text{)}}^{\text{2}}\times (0.05\text{)}\times (1-0.05)/{(\text{0.12)}}^{\text{2}}\\ & \text{= }66.69\\ & =\;67 \end{array}$$

where,

n = sample size
p = prevalence of acute poisoning,50%
e = margin of error, 12%
Z = 1.96 at 95% Confidence Interval

The calculated sample size was 67. Taking 13.5% non-response rate, total sample size is 76. Thus the 76 patients meeting inclusion criteria were enrolled in the study.

Written consent was obtained from participants. Epidemiological factors like age, gender, educational status, occupation, marital status, religion, were studied. Reason, motive and place to take poison, time elapse in the presentation to the hospital were also included in the study. Types of poison consumed and the route of administration was also studied. A convenient sampling method was applied.

The collected data were analyzed in the Statistical Package of the Social Sciences version 20 by using descriptive statistics. Point estimate at 95% Confidence Interval was calculated along with frequency and proportion for binary data.

## RESULTS

Out of 76 cases of acute poisoning, organophosphorus poisoning was 18 (23.7%) followed by unknown 12 (15.8%) and aluminum phosphide 7 (9.2 %) (Table 1).

**Table 1:** Type of poison.

Variables	n (%)
Alcohol	1 (1.3)
Aluminum Phosphide	7 (9.2)
Amitriptyline	2 (2.6 )
Battery liquid	1 (1.3)
Carbamate	2 (2.6)
Cough syrup	1 (1.3)
Fumes of chloride powder	1 (1.3)
Herbicides	1 (1.3)
Households chemicals	2 (2.6)
Ibuprofen	2 (2.6)
Insecticides	1 (1.3)
Metaacid	2 (2.6)
Methyl Spirit	4 (5.3)
Mosquito Repellant	1 (1.3)
Mushroom	1 (1.3)
Organophosphorus	18 (23.7)
Paracetamol	4 (5.3)
Phenol	2 (2.6)
Povidine iodine	1 (1.3)
Silica gel	1 (1.3)
Spirit	1 (1.3)
Turpentine Oil	1 (1.3)
Unknown	12 (15.8)
Urea	1 (1.3)
Wild honey	3 (3.9)
Zinc Phosphide	3 (3.9)
Total	76 (100)

The route of poison administration for oral was 72(94.7%) followed by inhalation 4 (5.3%). The reason to take the poison was Quarrel 28 (36.8%) followed byaccidental intake number 23(30.3%) (Table 2).

**Table 2:** Reason for taking poison.

Variables	n (%)
Accidental	23 (30.3)
Alcoholic	1 (1.3)
Anger	1 (1.3)
Enjoyment	1 (1.3)
Family pressure	2 (2.6)
Not disclosed	8 (10.5)
On grief	1 (1.3)
Quarrel	28 (36.8)
Relationship	5 (6.6)
Stupefying	2 (2.6)
Suicidal	3 (3.9)
Unknown	1 (1.3)
Total	76 (100)

Similarly, 41 (53.9%) had intention poisoning whereas 22 (28.9%) were unintentional. (Table 3).

**Table 3:** Motive for taking poison.

Variables	n (%)
Enjoyment	1 (1.3)
Family pressure	1 (1.3)
Not disclosed	2 (2.6)
Relationship	1 (1.3)
Stupefying	2 (2.6)
Suicidal	41 (53.9)
Threat	5 (6.6)
Unintentional	22 (28.9)
Unknown	1 (1.3)
Total	76 (100)

The average elapsed time to the visit of the emergency department was 110±80 minutes and out of 76, 15 (19.7%) took a poison on tuesday followed by Wednesday 12 (15.8%). (Table 4).

**Table 4:** Day of poisoning.

Variables	n (%)
Friday	10 (13.2)
Monday	12 (15.8)
Saturday	8 (10.5)
Sunday	10 (13.2)
Thursday	9 (11.8)
Tuesday	15 (19.7)
Wednesday	12 (15.8)
Total	76 (100)

The mean age of the patients was 26.9±15.2 years with minimum age of 15 months and maximum age of 70 years. Majority of the patients were female 45 (59.2%) and married 47 (61.8%). Regarding ethinic categroy, majority were from janajati hill 37 (48.7%), followed by khas 26 (34.2%), newar 6 (7.9%), khas (dalit) 4 (5.3%), madeshi 2 (2.6%), and muslim 1(1.3%). Majority were from hindu religion 44 (57.9%), followed by buddhist 29 (38.2%), christian 1 (2.6 %), and islam 1 (1.3%). Out of 76 patients, 19 (25%) were students, 16 (21.1%) were housewife, 10 (13.2%) were farmers, 10 (13.2%) were on menial class job, 7 (9.2%) were service holders, 6 (7.9%) were businessmen, 2 (2.6%) were unemployed, and remaining 6 (7.9%) were unknown. Sixteen (21.1%) were illiterate, and majority have completed primary level of education 29 (38.2%) followed by secondary level of education 10 (13.2%), intermediate 6 (7.9%), bachelors 6 (7.9%), SEE 4 (5.3%), plus two 4 (5.3%), Masters 1 (1.3%). The mean income of the patients was NRs. 9880±1648.65. Most of them were from nuclear families 61 (80.3%). Out of 76, only 4 (5.2%) had comorbid disease conditions.

Sixty-seven (88.2%) took a poison at home. Out of 76, 49 (64.5) them obtained a poison from home (Table 5).

**Table 5:** Place of obtaining poison.

Variables	n (%)
Farm	1 (1.3)
Friends	5 (6.6)
Home	49 (64.5)
Market	15 (19.7)
Medical Shop	6 (7.9)

## DISCUSSION

In our study, the mean age was found to be 26.9 years while in the study done in Chitwan, Nepal the mean age of the patients was 29.9 years.^6^ There is a predominance of the female gender (male:female : 1:1.4) in our study which was similar to the other study done in Nepal.^6,7,8,10^ Women are more prone to suicide attempt^6^ and are likely to be engaged in an impulsive act of self-harm.^9^

The majority were from Janajati hills and were Hindu followed by Buddhists which was similar to the study done in the past at the same site.^10^ However, various studies have concluded that social disadvantage, economically backward, and inequalities are associated with suicides.^6^

Most of them were students followed by housewives and farmers which was similar to the other study^6,7,11^ done in Nepal. Student and career beginners face pyscho-emotional problems involving failure in academics, financial hardship, family pressure, unemployment which may contribute towards a negative outlook of life.^6^

The mean income of the patient in our study was around NRs. 10,000 /months. In our study, illiterate and primary level of education contributed 59.3% of total patients which was similar to the study done by Shakya RP, et al.^7^ Negative thoughts towards life are more prone in illiteracy because lack of education contribute to less problem-solving approach.^7^

Comorbid disease condition didn't contribute much in poisoning as there were only 4 cases out of 76 cases. Also, most of them took poison at their own home and the source of obtaining poison was also home which was similar to the study done by Gyenwali D, et al.^6^ Another study was done by Shakya RP also concluded that 61.53% of the cases had poison stored in the home.^7^ Few studies have also concluded that suicide thought might have triggered by easy access to pesticides at home.^12,13^

Quarrel was the most common reason for poison in our study and a similar study was done in the past.^7^ The most common intention to take poison was suicide. This finding was similar to the study done in the same hospital in the past^10^ and other studies done in Nepal as well.^7,8,11^

The most common route was oral which was similar to the study done in other hospitals in Nepal.^11^ The Organophosphorus was the most common poison. Other studies done in Nepal have also concluded a similar result.^7,10,11,14^ It might be because pesticides and rodenticide are easily available in general convenience stores in Nepal.

After exposure to the poison, the mean time elapsed between exposure and arrival at the hospital was 1.8 hours and the most incidence of poisoning in our study happened on Tuesday. In one of the studies done in Nepal, the median time elapsed between exposure and arrival at the hospital was 2 hours.^6^

## CONCLUSIONS

The most common poisoning was organophosphorus with a suicide as a most common intention. The common reason to take poison was quarrel. Home was both the most frequent place to take and obtain poison. The mean time elapsed between exposure and arrival at the hospital was almost 2 hours.

Hospital-based single-center study and limited sample size should be considered as the limitation of this study. Therefore, a multi-center study with a large sample size should be considered as these kind of data provide preliminary important information regarding poison which may be useful tools to initiate preventive measures and formulate practical guidelines in the management of acute poisoning.
